# Significance of Increased Leptin Expression in Osteoarthritis Patients

**DOI:** 10.1371/journal.pone.0123224

**Published:** 2015-04-20

**Authors:** Ping Zhang, Zhi-Hong Zhong, Hao-Tao Yu, Bin Liu

**Affiliations:** 1 Department of Orthopedics, The Third Affiliated Hospital of Guangzhou Medical University, Guangzhou, 510000, P.R. China; 2 Department of Orthopedics, The Third Affiliated Hospital, Sun Yat-Sen University, Guangzhou, 510000, P.R. China; University of Modena & Reggio Emilia, ITALY

## Abstract

**Objective:**

Alterations in leptin expression contributes to the progression of various diseases, including cancers. This meta-analysis investigated the clinical significance of leptin levels in osteoarthritis (OA) patients, with the goal of building a leptin-based diagnostic criterion for OA.

**Method:**

Multiple scientific databases in English and Chinese languages, such as the Cochrane Library Database, CINAHL, Chinese Biomedical (CBM), EMBASE, PubMed, and Web of Science, were exhaustively searched, without any language restrictions, to identify high-quality studies relevant to leptin and OA. Version 12.0 STATA software was used for data analysis. We used odds ratios (OR) and 95% confidence intervals (CI) to test the correlation between serum leptin levels and OA progression.

**Results:**

A total of 11 clinical studies were finally selected for their high quality and relevance to the topic in this meta-analysis. The 11 case-control studies contained a combined total of 3,625 subjects. The meta-analysis results showed that leptin expression was significantly increased in OA patients, compared with the controls (SMD = 0.87, 95%CI: 0.72-1.02, *P* < 0.001), and there was also a strong association between leptin expression levels and gender (SMD = 8.55, 95%CI: 4.74-12.35, *P* < 0.001). In ethnicity-stratified subgroup analysis, all the study populations, irrespective of ethnicity, showed remarkably high leptin expression levels in females and in OA patients (all *P* < 0.05), compared to their respective counterparts.

**Conclusion:**

The present study revealed that increased leptin expression levels are associated with disease severity in OA patients, especially among the female OA patients. Based on our results, we propose that leptin level may be a useful biomarker for the assessment of the clinical status in OA patients.

## Introduction

Osteoarthritis (OA) is the most prevalent type of arthritis and is a painful degenerative joint disease involving cartilage and surrounding tissues, with a high incidence among elderly populations, especially among older females [1]. OA prevalence is approximately 40% in adults aged 70 and over, and is the major cause of pain as well as disability in this age group, particularly in women [2]. OA is mainly caused by an imbalance in the equilibrium between breakdown and repair of joint tissues, resulting in aberrant repair or insufficient self-repair of injured synovial joint tissues [3]. OA is characterized by consistent focal damage in the articular cartilage, as well as mild synovitis, muscle weakness, meniscal tears, and appositional new bone formation [4]. In addition, remodeling of osteophytes, sclerosis of the growth plate and subchondral trabeculae are also typically observed in OA. Due to the multi-factorial nature of the underlying OA pathogenesis, the etiology of OA is suspected to involve a complex interaction between the environmental factors and intrinsic risk factors [5]. The environmental factors include obesity, gender, mechanical stress, age, and joint trauma, with all of these playing pivotal roles in OA origin, but obesity appears to be a common and an essential component in OA pathogenesis [5–7]. Several previous studies have addressed the correlation between OA and pro-inflammatory mediators, and proposed a role for leptin, which is closely correlated with obesity, as a biomarker in OA [4,8].

Leptin is a ubiquitous 16-kDa pleiotropic protein primarily secreted by placenta or white adipose tissue [9]. Leptin controls adipose tissue volumes and body mass index (BMI) through regulating food intake and stimulating energy expenditure at the hypothalamus level, as a negative feedback loop at the hypothalamic nuclei [6,10]. Leptin is implicated in regulation of female endocrine reproductive system, and other biological pathways linked to immune responses, inflammatory diseases, cardiovascular functions and in respiratory pathophysiology. With specific relevance to bone diseases, leptin plays an indispensible role as a regulator of bone growth via the induction of collagen synthesis, mineralization of bone, proliferation of osteoblast, as well as the stimulation of endochondral ossification [11]. However, in clinical practice, high level of leptin expression is found in many diseases, including Alzheimer disease, breast cancer, and coronary heart diseases [12,13]. Recent studies also reported that the expression of leptin is detected in high concentrations in OA cartilage compared to normal cartilage [14,15]. As such, high levels of leptin expression increased matrix metaloprotease-9, matrix metaloprotease-13, and nitric oxide expression, and caused adverse effects on chondrocyte metabolism, eventually resulting in OA [14]. Additional evidence from published studies showed that over-expressed leptin is directly correlated with the grade of OA, and higher expression of leptin reflected the higher grade of OA [16,17]. Based on these previous studies, leptin appears to be closely linked with OA, and leptin levels could potentially be a convenient biomarker for predicting the severity of OA [18,19]. However, a few studies failed to such a strong link between leptin and OA and presented contradictory results [16,20]. To address this issue, we conducted a meta-analysis to investigate the influence of leptin expression level in OA progression, and further evaluate the correlation between leptin concentrations and the severity of OA.

## Materials and Methods

### Search Strategy

Relevant studies were identified by a comprehensive search of the following scientific databases: Cochrane Library Database, CINAHL, the Chinese Biomedical Database (CBM), EMBASE, PubMed, and the Web of Science, according to the PRISMA guidelines (http://prisma-statement.org/, as shown in S1 PRISMA Checklist). Studies published prior to April 30, 2014, and which assessed the relationships between the change in leptin expression and OA, were retrieved using the search terms (“Osteoarthritis, Spine” or “Osteoarthritis, Knee” or “Osteoarthritis, Hip” or “Osteoarthritis” or “knee osteoarthritis” or “spine osteoarthritis” or “hip osteoarthritis” or “spinal osteoarthritis” or “lumbar osteoarthritis” or “coxarthrosis”), and (“Leptin” or “leptin” or “Obese Protein” or “Obese Gene Product” or “Ob Gene Product” or “Ob Protein”). No limitation was placed on the language of the article. Additionally, potential relevant articles were further retrieved through a manual search of cross-references from original reports.

### Selection Criteria

The study selection criteria were: (1) randomized intervention case-control studies or cohort studies that involved the association between expression levels of leptin and osteoarthritis as a primary outcome were included in this study, (2) included patients confirmed with the diagnosis of OA, in accordance with the definition and classification provided by the Diagnostic and Therapeutic Criteria Committee of the American Rheumatism Association [21], (3) associated with Leptin expression levels. The exclusion criteria were: (1) studies that did not provide the number of OA cases or sufficient information about serum leptin expression levels, (2) the minimum number of cases in included studies were less than 34, (3) duplicate publications, studies with incomplete data were excluded. Studies with the largest sample size and the most recent publication years were included when multiple studies published with the same author and with similar case series.

### Data Extraction

From each selected article, two investigators independently extracted the required information, using a standardized protocol and data recording form. Disagreements on data or study inclusion were resolved by discussion of all the items to reach a consensus. The extracted information, included surname of initial authors, publication years, source of publication, study type, study design, sample size, age, sex, ethnicity and country, source of controls, source of samples, detection method of leptin expression levels, and expression levels of leptin, was collected from each study prospectively. Due to subjects from different ethnicities, information was extracted separately and classified into Asians, Caucasians, and Mixed. All authors approved the final inclusion of studies for meta-analysis.

### Quality Assessment

To decide whether the study in question is of high quality, two authors used a set of predefined criterion based on the Newcastle-Ottawa Scale (NOS) criteria to assess the studies independently [22]. Three aspects were derived from the criteria of NOS: (1) subject selection: 0~4; (2) inter-subject comparability: 0~2; (3) clinical outcome: 0~3. The total score degrees range from 0 (lowest) to 9 (highest). According to the NOS scores, the included studies were classified into two levels: low quality (0–6), and high quality (7–9), respectively. The NOS quality assessment scales are presented in in S1 NOS Quality Assessment. A third reviewer was consulted when disagreements or discrepancies between the two investigators existed with regard to the NOS scores of the studies.

### Statistical Analysis

In this meta-analysis, we used random-effects model or a fixed-effects model in situations where data from independent studies were combined to provide quantitative evidence and to minimize the variance of the summaries. A random-effect model was used when heterogeneity existed among studies, or the fixed-effects model was used in cases where no statistically significant heterogeneity was observed. The summary standardized mean difference (SMD) with 95% confidence intervals (CIs) was calculated for case versus control and female versus male categories of leptin expression levels by *Z* test. The subgroup meta-analyses were also conducted by ethnicity and source of sample to explore potential effect modification. Heterogeneity across the enrolled studies was evaluated by the Cochran’s *Q*-statistic (*P* < 0.05 was regarded as statistically significant) [23]. As a result of low statistical power of the Cochran’s *Q*-statistic, *I* ^*2*^ test was also measured to reflect the possibility of heterogeneity between studies [24]. The sensitivity analysis evaluated whether the results could have been influenced significantly by any one single study, by deleting each study one by one. The funnel plot was constructed to assess how publication bias might affect the validity of the estimates. The symmetry of the funnel plot was further evaluated by Egger's linear regression test [25]. All tests were two-sided and a *P* value of < 0.05 was considered as statistically significant. To ensure credible and accurate results, investigators separately input all the data into the STATA software, version 12.0 (Stata Corp, College Station, TX, USA) and arrived at a final decision.

## Results

### Baseline Characteristics of Extracted Studies

**Fig 1:**
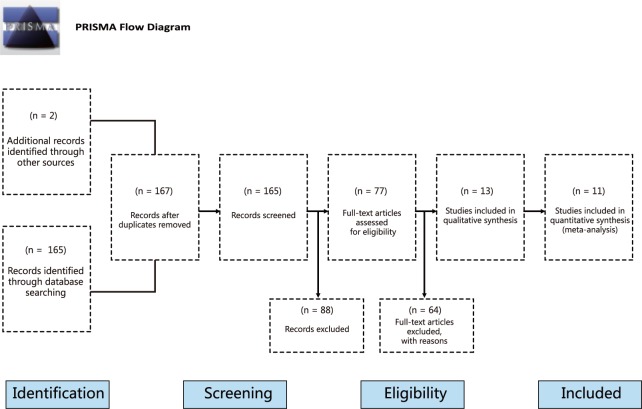
Flow chart of literature search and study selection. Eleven case-control studies were included in this meta-analysis.

We initially retrieved a total of 167 studies from the scientific literature databases search and the flow chart of study selection process is summarized in Fig 1. By screening the title, key words and abstracts, 88 of these studies were excluded for various reasons (2 were duplicates, 25 were letters, reviews or meta-analyses, 29 were non-human studies, and 32 were not related to the research topics). Full-text articles from the remaining 79 articles were re-examined and another 66 trials were excluded (19 were not case-control, 22 were not relevant to leptin, and 25 were not relevant to OA), leaving 13 studies to be considered in the next screening step. Of these, 2 were excluded for not providing sufficient data, and finally 11 studies published between 2007 and 2014 [5,7,16,18–20,26–30], and including a total of 3,625 subjects, conformed to our stringent inclusion criteria. The search outcomes are presented as S1 Search Outcomes. Among the studies included in this meta-analysis, 2 studies were in Asian populations, 6 were in Caucasian populations, and 3 studies involved mixed populations. Detection methods used in the included studies for detection of leptin levels were enzyme linked immunosorbent assay (ELISA) and radioimmunoassay (RIA). Sources of tissue samples for leptin levels in studies included in our present meta-analysis were blood, plasma, and synovial fluid (SF). Quality scores of the enrolled papers were higher than 7 (high quality). Tables 1 and 2 summarize the characteristics and the quality of methodological of all the enrolled studies.

**Table 1:** Characteristics of included studies focused on protein expression of LEP.

First author	Year	Ethnicity	Number		Age (years)		Sample	Method	NOS score
			Male	Female	Male	Female			
Perruccio AV[27]	2014	Caucasians	33	45	61.4 ± 11.3	64.7 ± 10.1	Plasma	ELISA	7
Staikos C[5]	2013	Caucasians	21	75	64.4 ± 10.4		Plasma	ELISA	7
					64.4 ± 10.4		SF	ELISA	
Massengale M[19]	2012	Caucasians	510	546	-	-	Plasma	RIA	8
Karvonen-Gutierrez CA[18]	2012	Mix	517	554	69.9 ± 0.16	71.0 ± 0.16	Plasma	ELISA	8
Stannus OP[7]	2010	Caucasians	100	93	63.3 ± 7.2	61.9 ± 6.9	Plasma	RIA	8
Ku JH[16]	2009	Asians	15	27	-	-	SF	ELISA	6
Gegout PP[26]	2008	Caucasians	20	15	65.80 ± 7.74	71.05 ± 7.41	SF	ELISA	6
Wang ZH[20]	2007	Asians	24	43	64 ± 17.2		Plasma	RIA	7

Legends: ELISA—enzyme linked immunosorbent assay, RIA—radioimmunoassay, NOS—Newcastle-Ottawa Scale

**Table 2:** Characteristics of included studies focused on protein expression of LEP.

First author	Year	Ethnicity	Sample	Sample size		Gender (M/F)		Age (years)		Method	NOS score
				Case	Control	Case	Control	Case	Control		
Karvonen-Gutierrez CA[18]	2013	Mixed	Blood	98	444	-	-	46.0 ± 2.6	46.1 ± 2.8	ELISA	7
Beekhuizen M[28]	2012	Mixed	SF	18	16	172/262	340/292	71.7 ± 0.1	69.8 ± 0.1	ELISA	6
de Boer TN[29]	2012	Caucasians	Blood	172	132	53/119	34/98	67.4 ± 8.4	56.5 ± 4.5	ELISA	8
Ku JH[16]	2009	Asians	SF	42	10	15/27	7/3	62.1 ± 10.5	36.4 ± 11.4	ELISA	6
Wang ZH[20]	2007	Asians	Blood	67	30	24/43	11/19	64.0 ± 17.2	63.0 ± 13.3	RIA	6

Legends: M—male, F—female, ELISA—enzyme linked immunosorbent assay, RIA—radioimmunoassay, NOS—Newcastle-Ottawa Scale

### Expression levels of Leptin in Osteoarthritis

**Fig 2:**
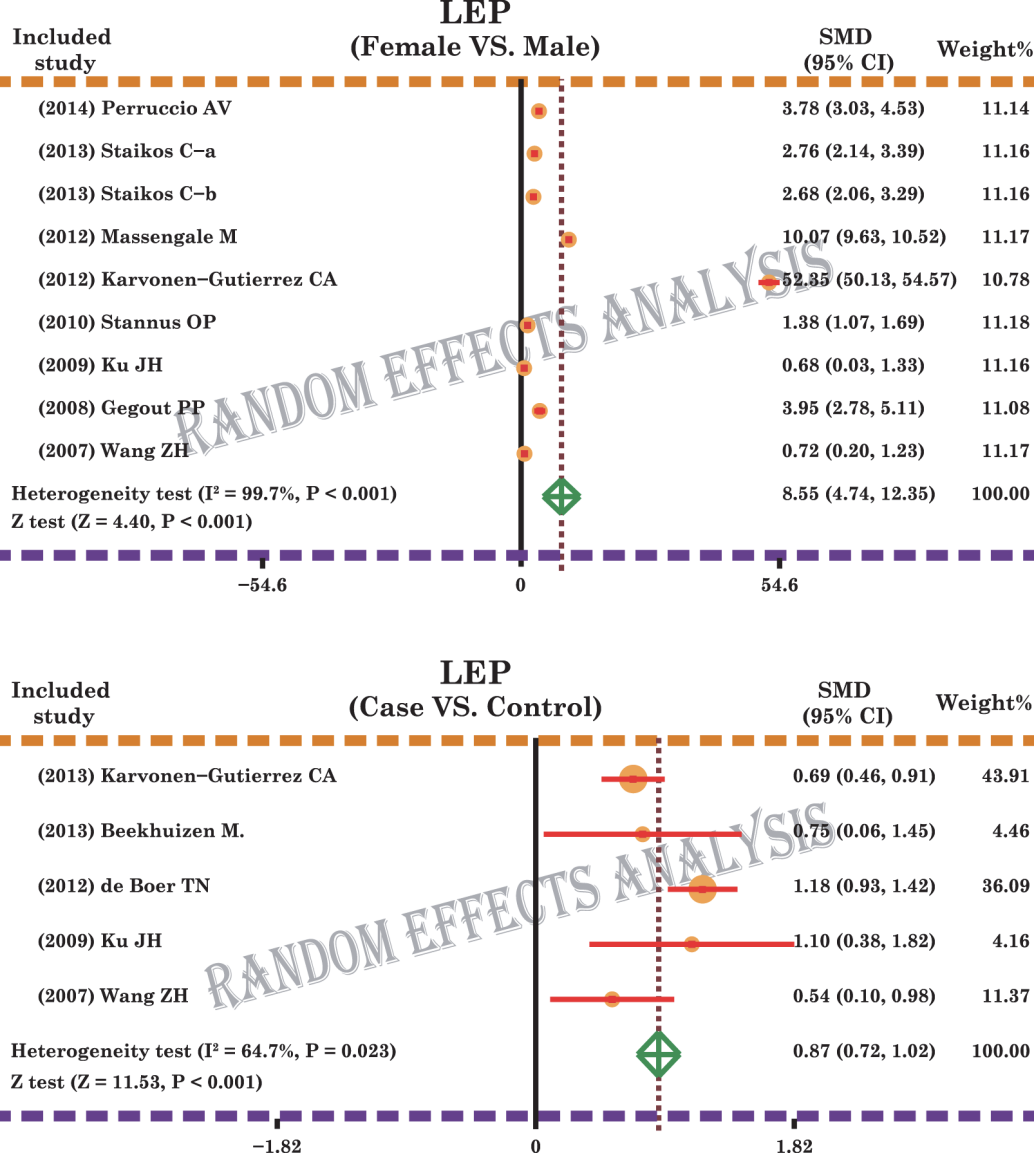
Forest plot for the relationships between leptin expression levels and the development of osteoarthritis.

**Fig 3:**
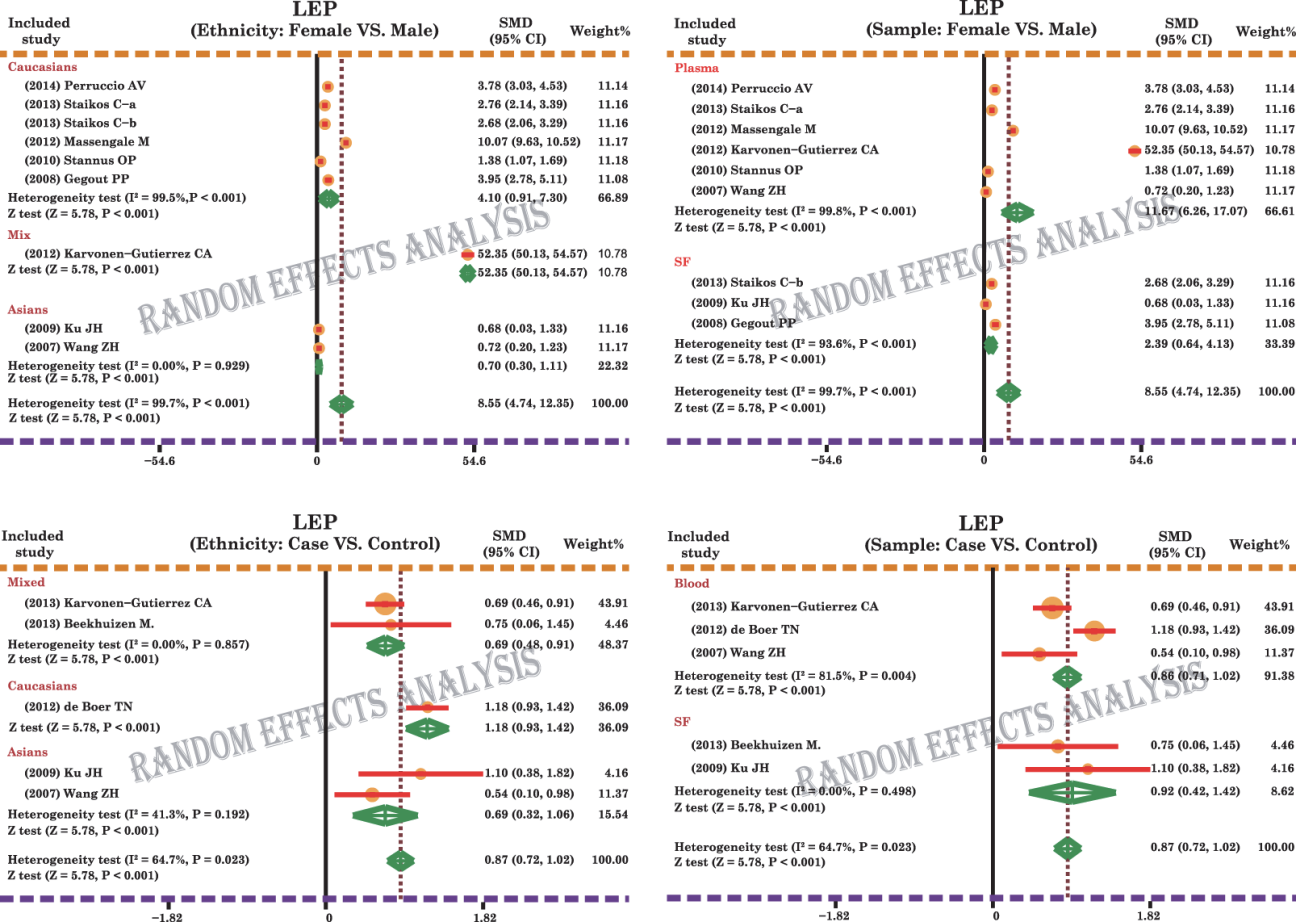
Subgroup analyses for the relationships between leptin expression levels and the development of osteoarthritis.

A total of 11 case-control and cohort studies measured the leptin expression levels in OA. The major results of the correlations between the expression levels of leptin and OA are shown in Fig 2. The random-effects model was used because of existing heterogeneity (both *P* < 0.05). Meta-analysis results identified a positive association between leptin expression levels and OA (SMD = 0.87, 95%CI: 0.72–1.02, *P* < 0.001), and females showed a significantly higher correlation of leptin levels with OA, compared to no such correlation observed in males (SMD = 8.55, 95%CI: 4.74–12.35, *P* < 0.001). Subgroup analysis based on ethnicity revealed that higher leptin level in females, compared with males, was observed in Asian, Caucasian and Mixed populations, and thus was independent of ethnic differences (all *P* < 0.05). In addition, our results showed that high level of leptin expression is a significant risk factor for OA in Asians, Caucasians and Mixed populations (all *P* < 0.05), again reinforcing that leptin levels correlated with OA independent of potential ethnic differences (Fig 3). Sample-stratified analysis displayed that leptin expression levels were correlated to female in both Plasma and SF subgroups, as seen in Fig 3 (Plasma: SMD = 11.67, 95%CI: 6.26–17.07, *P* < 0.001; SF: SMD = 2.39, 95%CI: 0.64–4.13, *P* < 0.001; respectively). Further analysis by the source of samples also showed positive associations between the expression levels of leptin and OA in both blood and SF subgroups (Blood: SMD = 0.86, 95%CI: 0.71–1.02, *P* < 0.001; SF: SMD = 0.92, 95%CI: 0.42–1.42, *P* < 0.001; respectively).

### Sensitivity Analysis and Publication Bias

**Fig 4:**
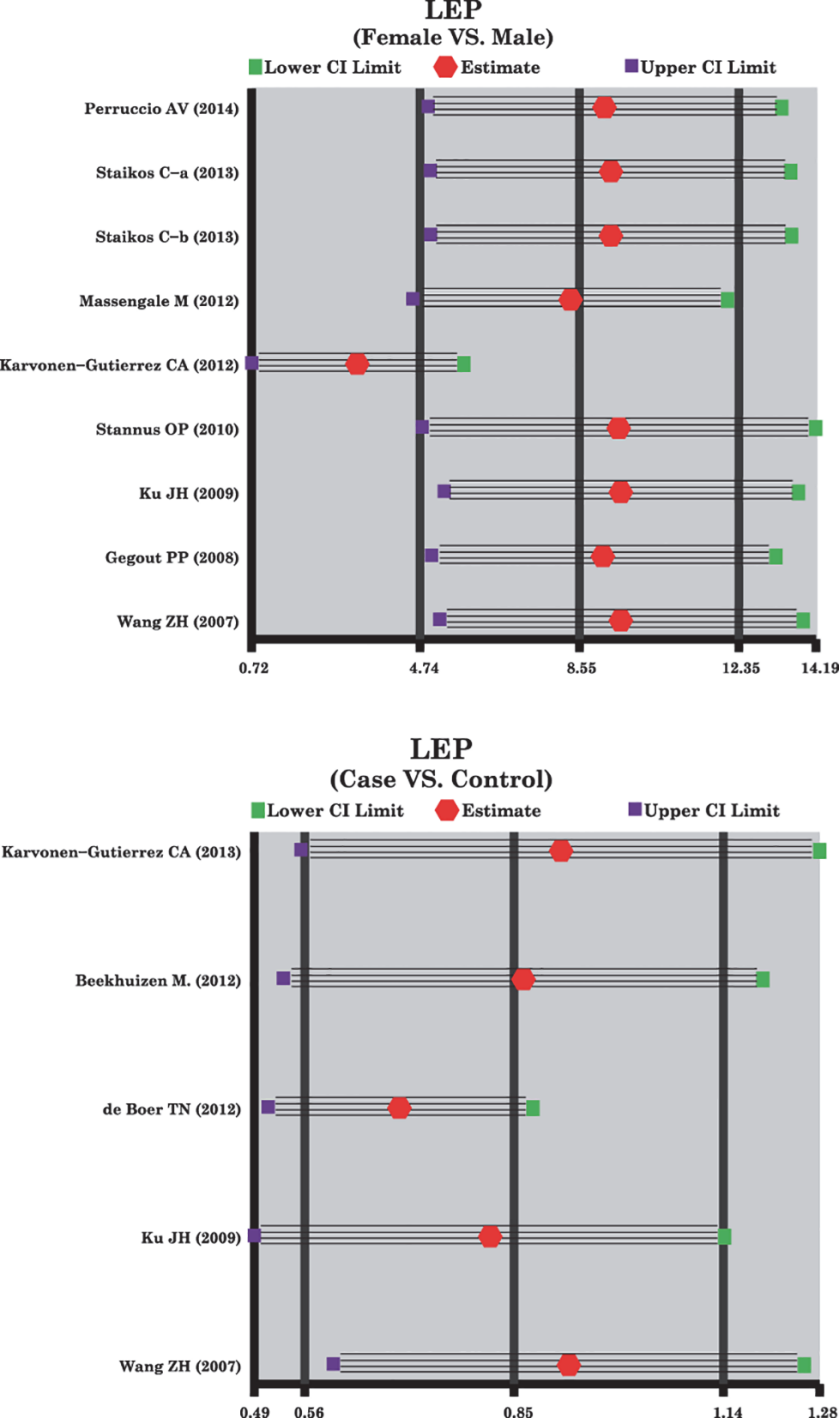
Sensitivity analysis, to evaluate whether the overall results regarding the relationships between leptin expression levels and the development of osteoarthritis could have been significantly influenced by one single study, was performed through deleting individual studies one by one.

**Fig 5:**
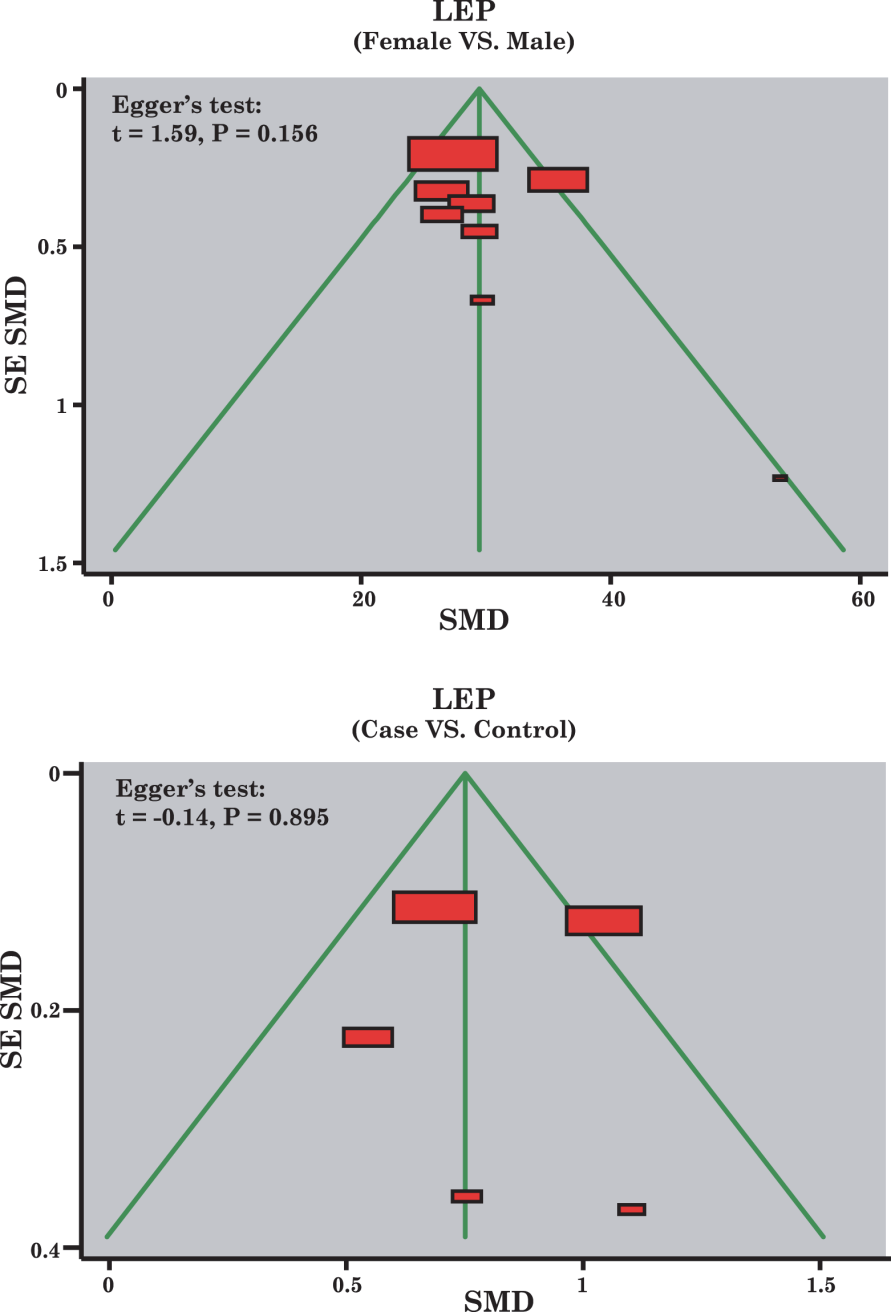
Funnel plot of publication biases on the relationships between leptin expression levels and the development of osteoarthritis.

A sensitivity analysis was carried out to evaluate whether the present meta-analysis is stable, and results showed that the overall statistical significance does not change when any single study was omitted. Therefore, the current meta-analysis data is relatively stable and credible (Fig 4). The graphical funnel plots of those 11 studies present symmetrical, and Egger's test showed that there is no publication bias in this meta-analysis (all *P* > 0.05) (Fig 5).

## Discussion

In our study, we found up-regulated expression levels of leptin in OA patients compared to healthy subjects, implying that alterations in leptin expression may be related to OA, thus the overexpression of leptin may be a risk factor in OA. Furthermore, the findings in our study implicated that leptin expression level was higher in female OA patients than male OA patients, suggesting significant gender differences. Thus, we propose that leptin levels may be a very sensitive biomarker for OA among women, and more focused studies in the future addressing this issue may uncover interesting clinical applications of leptin. Nevertheless, the precise mechanism by which leptin expression levels are linked with the progression and the outcomes in OA is not fully understood. OA is characterized by a deterioration and progressive loss of articular cartilage [31]. Obesity combined with aging and genetic factors are recognized as important risk factors for OA [32]. In addition, leptin combined with proinflammatory cytokines, including interleukins and matrix metalloproteinase, may also have a potential influence on cartilage metabolism [6,10]. It has been reported that the serum leptin levels are higher in OA cartilage compared to normal cartilage, and also that the leptin expression levels are linked with the severity of cartilage impairment [33]. As stated above, leptin exerts mainly pro-inflammatory and catabolic activities in osteoarthritic joints, in agreement with the fact that the level of leptin expression was elevated in OA patients. On the other hand, we found that females have both higher circulating leptin levels and a greater propensity to develop OA compared with males [34, 35]. It is possible that estradiol may promote the secretion of leptin in female patients while testosterone may inhibit the leptin secretion in men. Consistent with our results, Karvonen-Gutierrez et al. also observed that the leptin expression level was related to the pathogenesis of OA among men and women, and the higher level of leptin appeared to be important in female OA patients as a potential risk factor [18].

To investigate the relationship between the leptin expression level and OA progression, we performed stratified analysis on the basis of ethnicity and source of sample. Our findings of ethnicity-stratified analysis showed that high leptin expression was linked with an increased risk of OA among Caucasians, Asians and mixed populations. Among different ethnicities, our subgroup analysis results showed that leptin level among female patients with OA was higher than male OA patients in Caucasians, Asians and mixed populations, indicating that ethnicity differences may not influence this outcome. Further subgroup analysis based on the source of sample showed that there was a positive connection between enhanced expression of leptin and the osteoarthritis development in both blood and SF subgroups. We have also found that leptin expression levels were correlated to gender in both plasma and SF subgroups, suggesting that different sample sources were not the potential heterogeneity source of this outcome. In summary, consistent with previous studies, our results indicate that the level of leptin expression was significantly up-regulated in OA, especially in female patients, implying that overexpression of leptin may relate to the occurrence and progression of OA. We also show that leptin levels are clearly influenced by and affect the severity of OA, thus can be used as a potential biomarker of OA in specific clinical settings.

Our meta-analysis also had several limitations. First, owing to a small sample size in this meta-analysis, some necessary information could not be obtained. Second, possible publication bias could exist, though no indication of publication bias was detected in the Egger's test. Third, studies extracted in this meta-analysis are comprised of diverse ethnic populations and nations, as well as genders, lifestyles, ages, and cultures. In addition, differences in access to healthcare and efficacy judgments could also influence the severity of the disease and the data collected. Fourth, we included English or Chinese studies and excluding studies in other languages may be a potential selection bias. Fifth, case control and cohorts designed studies were included for meta-analyses, which might result in heterogeneity, and no other subgroup analyses based on study design were performed in our stratified analysis, which may affect the credibility and reliability of the present results. More importantly, meta-analysis of aggregate observational data produces estimates of effect that are unadjusted for confounding and interactions and as such these need to be interpreted with caution. Finally, several explanatory variables were not included in this meta-analysis, especially some environmental factors such as movement, activity or “wear and tear”, which might affect the reliability and credibility of our results to some extent. Despite these limitations, our study is the first meta-analysis to investigate the correlation between leptin expression level and OA development.

In conclusion, the current meta-analysis revealed that up-regulation of leptin levels was distinctly correlated with the progression of disease in OA patients, especially among female OA patients. Leptin may, thus, be an important biomarker in the clinic for monitoring progression of the disease or as a confirmatory marker of OA in early stages of the disease. Importantly, a meta-analysis using individual patient data is required for more precise estimates of observed effects. Further clinical research is needed to investigate the precise clinical significance of leptin and to determine whether leptin levels predict clinical prognosis and outcomes in OA patients.

## Supporting Information

S1 PRISMA Checklist The PRISMA 2009 checklist. (DOC)

S1 NOS Quality Assessment The Newcastle-Ottawa Scale for assessing methodological quality. (DOCX)

S1 Search Outcomes The electronic searching outcomes. (DOC)
