# Supplementary material for: Significance of Increased Leptin Expression in Osteoarthritis Patients
Source: PLoS One. 2015 Apr 20;10(4):e0123224. doi: 10.1371/journal.pone.0123224 (PMC4403877; doi:10.1371/journal.pone.0123224)
Supplement: S1 NOS Quality Assessment — (DOCX) [file pone.0123224.s002.docx]

**S1 – S1 The electronic searching outcomes.**

(1) Review, case report, meta-analysis: 25

1. Zhen J, Guo HY, Pan SJ, Zhang PF, et al. (2014) Progress of adipocytokines and osteoarthritis research. Basic medical sciences and clinics 34(4): 562-565.

2. Stannus OP, Jones G, Quinn SJ, Cicuttini FM, Dore D, et al. (2010) The association between leptin, interleukin-6, and hip radiographic osteoarthritis in older people: a cross-sectional study. Arthritis Res Ther 12(3): R95

3. Hu PF, Bao JP, Wu LD (2011) The emerging role of adipokines in osteoarthritis: a narrative review. Mol Biol Rep 38(2): 873-878.

4. Qin J, Shi D, Dai J, Zhu L, Tsezou A, et al. (2010) Association of the leptin gene with knee osteoarthritis susceptibility in a Han Chinese population: a case-control study. J Hum Genet 55(10): 704-706.

5. Stannus OP, Jones G, Quinn SJ, Cicuttini FM, Dore D, et al. (2010) The association between leptin, interleukin-6, and hip radiographic osteoarthritis in older people: a cross-sectional study. Arthritis Res Ther 12(3): R95

6. Zhen J, Guo HY, Pan SJ, Zhang PF, et al. (2014) Progress of adipocytokines and osteoarthritis research. Basic medical sciences and clinics 34(4): 562-565.

7. Miller GD, Jenks MZ, Vendela M, Norris JL, Muday GK (2012) Influence of weight loss, body composition, and lifestyle behaviors on plasma adipokines: a randomized weight loss trial in older men and women with symptomatic knee osteoarthritis. J Obes 2012: 708505.

8. Oren TW, Botolin S, Williams A, Bucknell A, King KB (2011) Arthroplasty in veterans: analysis of cartilage, bone, serum, and synovial fluid reveals differences and similarities in osteoarthritis with and without comorbid diabetes. J Rehabil Res Dev 48(10): 1195-1210.

9. Presle N, Pottie P, Dumond H, Guillaume C, Lapicque F, et al. (2006) Differential distribution of adipokines between serum and synovial fluid in patients with osteoarthritis. Contribution of joint tissues to their articular production. Osteoarthritis Cartilage 14(7): 690-695.

10. Schaffler A, Ehling A, Neumann E, Herfarth H, Tarner I, et al. (2003) Adipocytokines in synovial fluid. JAMA 290(13): 1709-1710.

11. Staikos C, Ververidis A, Drosos G, Manolopoulos VG, Verettas DA, et al. (2013) The association of adipokine levels in plasma and synovial fluid with the severity of knee osteoarthritis. Rheumatology (Oxford) 52(6): 1077-1083.

12. Chen HH, Wu ZQ, Gao DW, Wu YF, (2012) Clinical study on Bushen Jianxi Decoction in the treatment of knee osteoarthritis with osteoporosis. Yiayao Qianyan 02(17): 330-331.

13. Pallu S, Francin PJ, Guillaume C, Gegout-Pottie P, Netter P, et al. (2010) Obesity affects the chondrocyte responsiveness to leptin in patients with osteoarthritis. Arthritis Res Ther 12(3): R112.

14. Teichtahl AJ, Wluka AE, Proietto J, Cicuttini FM (2005) Obesity and the female sex, risk factors for knee osteoarthritis that may be attributable to systemic or local leptin biosynthesis and its cellular effects. Med Hypotheses 65(2): 312-315.

15. Vuolteenaho K, Koskinen A, Kukkonen M, Nieminen R, Paivarinta U, et al. (2009) Leptin enhances synthesis of proinflammatory mediators in human osteoarthritic cartilage--mediator role of NO in leptin-induced PGE2, IL-6, and IL-8 production. Mediators Inflamm 2009(345838.

16. Vuolteenaho K, Koskinen A, Moilanen T, Moilanen E (2012) Leptin levels are increased and its negative regulators, SOCS-3 and sOb-R are decreased in obese patients with osteoarthritis: a link between obesity and osteoarthritis. Ann Rheum Dis 71(11): 1912-1913.

17. Stannus OP, Cao Y, Antony B, Blizzard L, Cicuttini F, et al. (2013) Cross-sectional and longitudinal associations between circulating leptin and knee cartilage thickness in older adults. Ann Rheum Dis.

18. Tong KM, Shieh DC, Chen CP, Tzeng CY, Wang SP, et al. (2008) Leptin induces IL-8 expression via leptin receptor, IRS-1, PI3K, Akt cascade and promotion of NF-kappaB/p300 binding in human synovial fibroblasts. Cell Signal 20(8): 1478-1488.

19. Toussirot E, Streit G, Wendling D (2007) The contribution of adipose tissue and adipokines to inflammation in joint diseases. Curr Med Chem 14(10): 1095-1100.

20. Schaffler A, Ehling A, Neumann E, Herfarth H, Tarner I, et al. (2003) Adipocytokines in synovial fluid. JAMA 290(13): 1709-1710.

21. Chen HH, Wu ZQ, Gao DW, Wu YF, (2012) Clinical study on Bushen Jianxi Decoction in the treatment of knee osteoarthritis with osteoporosis. Yiayao Qianyan 02(17): 330-331.

22. Sandell LJ (2009) Obesity and osteoarthritis: is leptin the link? Arthritis Rheum 60(10): 2858-2860.

23. Jia P, Zhao WG, Wang YG, Zhang L, (2007) Transforming growth factor - β, leptin and osteoarthritis. Chin J Coal Industry Med 10(9): 993-995.

24. Chen J, Zhao WG, Cai LS, et al. (2008) Leptin and osteroarthritis. J North China Coal Medical University 10(2): 184-186.

25. Ma XJ, Guo HH, Hao SW, Sun SX, Yang XC, et al. (2013) Association of single nucleotide polymorphisms (SNPs) in leptin receptor gene with knee osteoarthritis in the Ningxia Hui population. 35(3): 359-364.

(2) Not human studies: 29

1. Griffin TM, Fermor B, Huebner JL, Kraus VB, Rodriguiz RM, et al. (2010) Diet-induced obesity differentially regulates behavioral, biomechanical, and molecular risk factors for osteoarthritis in mice. Arthritis Res Ther 12(4): R130.

2. Griffin TM, Guilak F (2008) Why is obesity associated with osteoarthritis? Insights from mouse models of obesity. Biorheology 45(3-4): 387-398.

3. Griffin TM, Huebner JL, Kraus VB, Guilak F (2009) Extreme obesity due to impaired leptin signaling in mice does not cause knee osteoarthritis. Arthritis Rheum 60(10): 2935-2944.

4. Griffin TM, Huebner JL, Kraus VB, Yan Z, Guilak F (2012) Induction of osteoarthritis and metabolic inflammation by a very high-fat diet in mice: effects of short-term exercise. Arthritis Rheum 64(2): 443-453.

5. Heep H, Hilken G, Hofmeister S, Wedemeyer C (2009) Osteoarthitis of leptin-deficient ob/ob mice in response to biomechanical loading in micro-CT. Int J Biol Sci 5(3): 265-275.

6. Simopoulou T, Malizos KN, Iliopoulos D, Stefanou N, Papatheodorou L, et al. (2007) Differential expression of leptin and leptin's receptor isoform (Ob-Rb) mRNA between advanced and minimally affected osteoarthritic cartilage; effect on cartilage metabolism. Osteoarthritis Cartilage 15(8): 872-883.

7. Chen HH, Wu ZQ, Gao DW, Wu YF, (2012) Clinical study on Bushen Jianxi Decoction in the treatment of knee osteoarthritis with osteoporosis. Yiayao Qianyan 02(17): 330-331.

8. Sandell LJ (2009) Obesity and osteoarthritis: is leptin the link? Arthritis Rheum 60(10): 2858-2860.

9. Jia P, Zhao WG, Wang YG, Zhang L, (2007) Transforming growth factor - β, leptin and osteoarthritis. Chin J Coal Industry Med 10(9): 993-995.

10. J, Zhao WG, Cai LS, et al. (2008) Leptin and osteroarthritis 10(2): 184-186.

11. Ma XJ, Guo HH, Hao SW, Sun SX, Yang XC, et al. (2013) Association of single nucleotide polymorphisms (SNPs) in leptin receptor gene with knee osteoarthritis in the Ningxia Hui population]35(3): 359-364.

12. Yang L, Guo A, (2010) Effect of leptin in the pathogenesis of osteoarthritis. Orthopedic J China 18(7): 562-564.

13. Simopoulou T, Malizos KN, Iliopoulos D, Stefanou N, Papatheodorou L, et al. (2007) Differential expression of leptin and leptin's receptor isoform (Ob-Rb) mRNA between advanced and minimally affected osteoarthritic cartilage; effect on cartilage metabolism. Osteoarthritis Cartilage 15(8): 872-883.

14. Yusuf E, Ioan-Facsinay A, Bijsterbosch J, Klein-Wieringa I, Kwekkeboom J, et al. (2011) Association between leptin, adiponectin and resistin and long-term progression of hand osteoarthritis. Ann Rheum Dis 70(7): 1282-1284.

15. Zhang ZM, Jiang LS, Jiang SD, Dai LY (2009) Osteogenic potential and responsiveness to leptin of mesenchymal stem cells between postmenopausal women with osteoarthritis and osteoporosis. J Orthop Res 27(8): 1067-1073.

16. Wang ZH, Zhang P, Jiao WX, Ren ZQ, Wang TJ, (2007) Determination and significance of knee osteoarthritis patients serum leptin levels Medical Information 20(7): 1222-1223.

17. Karvonen-Gutierrez CA, Sowers MR, Heeringa SG (2012) Sex dimorphism in the association of cardiometabolic characteristics and osteophytes-defined radiographic knee osteoarthritis among obese and non-obese adults: NHANES III. Osteoarthritis Cartilage 20(7): 614-621.

18. Katz JD, Agrawal S, Velasquez M (2010) Getting to the heart of the matter: osteoarthritis takes its place as part of the metabolic syndrome. Curr Opin Rheumatol 22(5): 512-519.

19. Magliano M (2008) Obesity and arthritis. Menopause Int 14(4): 149-154.

20. Massengale M, Lu B, Pan JJ, Katz JN, Solomon DH (2012) Adipokine hormones and hand osteoarthritis: radiographic severity and pain. PLoS One 7(10): e47860.

21. Lajeunesse D, Pelletier JP, Martel-Pelletier J (2005) Osteoarthritis: a metabolic disease induced by local abnormal leptin activity? Curr Rheumatol Rep 7(2): 79-81.

22. Miller GD, Nicklas BJ, Davis CC, Ambrosius WT, Loeser RF, et al. (2004) Is serum leptin related to physical function and is it modifiable through weight loss and exercise in older adults with knee osteoarthritis? Int J Obes Relat Metab Disord 28(11): 1383-1390.

23. Mutabaruka MS, Aoulad Aissa M, Delalandre A, Lavigne M, Lajeunesse D (2010) Local leptin production in osteoarthritis subchondral osteoblasts may be responsible for their abnormal phenotypic expression. Arthritis Res Ther 12(1): R20.

24. Ma XJ, Guo HH, Hao SW, Sun SX, Yang XC, et al. (2013) Association of single nucleotide polymorphisms (SNPs) in leptin receptor gene with knee osteoarthritis in the Ningxia Hui population. Yi Chuan 35(3): 359-364.

25. Niu SP, Huang CB, Zhao LK, Cheng YJ, Yang T (2011) The role of promoter CpG islands methylation of leptin gene in osteoarthritis. Zhonghua Nei Ke Za Zhi 50(1): 55-58.

26. Terlain B, Dumond H, Presle N, Mainard D, Bianchi A, et al. (2005) Is leptin the missing link between osteoarthritis and obesity?. Ann Pharm Fr 63(3): 186-193.

27. Terlain B, Presle N, Pottie P, Mainard D, Netter P (2006) Leptin: a link between obesity and osteoarthritis?. Bull Acad Natl Med 190(7): 1421-1435; discussion 1435-1427, 1475-1427.

28. Zborovskaia IA, Simakova ES, Zavodovskii BV, Akhverdian Iu R, Kononov VE (2013) Prognostic value of leptin level detection in industrial workers suffering from osteoarthrosis in Volgograd. Med Tr Prom Ekol1): 34-38.

29. Ku JH, Lee CK, Joo BS, An BM, Choi SH, et al. (2009) Correlation of synovial fluid leptin concentrations with the severity of osteoarthritis. Clin Rheumatol 28(12): 1431-1435.

(4) Not case control:19

1. Ma XJ, Guo HH, Hao SW, Sun SX, Yang XC, et al. (2013) Association of single nucleotide polymorphisms (SNPs) in leptin receptor gene with knee osteoarthritis in the Ningxia Hui population. Yi Chuan 35(3): 359-364.

2. Niu SP, Huang CB, Zhao LK, Cheng YJ, Yang T (2011) The role of promoter CpG islands methylation of leptin gene in osteoarthritis. Zhonghua Nei Ke Za Zhi 50(1): 55-58.

3. Terlain B, Dumond H, Presle N, Mainard D, Bianchi A, et al. (2005) Is leptin the missing link between osteoarthritis and obesity?. Ann Pharm Fr 63(3): 186-193.

4. Terlain B, Presle N, Pottie P, Mainard D, Netter P (2006) Leptin: a link between obesity and osteoarthritis?. Bull Acad Natl Med 190(7): 1421-1435; discussion 1435-1427, 1475-1427.

5. Zborovskaia IA, Simakova ES, Zavodovskii BV, Akhverdian Iu R, Kononov VE (2013) Prognostic value of leptin level detection in industrial workers suffering from osteoarthrosis in Volgograd. Med Tr Prom Ekol1): 34-38.

6. Karvonen-Gutierrez CA, Harlow SD, Mancuso P, Jacobson J, Mendes de Leon CF, et al. (2013) Association of leptin levels with radiographic knee osteoarthritis among a cohort of midlife women. Arthritis Care Res (Hoboken) 65(6): 936-944.

7. Ku JH, Lee CK, Joo BS, An BM, Choi SH, et al. (2009) Correlation of synovial fluid leptin concentrations with the severity of osteoarthritis. Clin Rheumatol 28(12): 1431-1435.

8. Wislowska M, Rok M, Jaszczyk B, Stepien K, Cicha M (2007) Serum leptin in rheumatoid arthritis. Rheumatol Int 27(10): 947-954.

9. Ku JH, Lee CK, Joo BS, An BM, Choi SH, et al. (2009) Correlation of synovial fluid leptin concentrations with the severity of osteoarthritis. Clin Rheumatol 28(12): 1431-1435.

10. Massengale M, Reichmann WM, Losina E, Solomon DH, Katz JN (2012) The relationship between hand osteoarthritis and serum leptin concentration in participants of the Third National Health and Nutrition Examination Survey. Arthritis Res Ther 14(3): R132.

11. Dumond H, Presle N, Terlain B, Mainard D, Loeuille D, et al. (2003) Evidence for a key role of leptin in osteoarthritis. Arthritis Rheum 48(11): 3118-3129.

12. Durmus D, Alayli G, Aliyazicioglu Y, Buyukakincak O, Canturk F (2013) Effects of glucosamine sulfate and exercise therapy on serum leptin levels in patients with knee osteoarthritis: preliminary results of randomized controlled clinical trial. Rheumatol Int 33(3): 593-599.

13. Fioravanti A, Cantarini L, Bacarelli MR, de Lalla A, Ceccatelli L, et al. (2011) Effects of spa therapy on serum leptin and adiponectin levels in patients with knee osteoarthritis. Rheumatol Int 31(7): 879-882.

14 Gandhi R, Takahashi M, Syed K, Davey JR, Mahomed NN (2010) Relationship between body habitus and joint leptin levels in a knee osteoarthritis population. J Orthop Res 28(3): 329-333.

15. Gualillo O (2007) Further evidence for leptin involvement in cartilage homeostases. Osteoarthritis Cartilage 15(8): 857-860.

16. Koskinen A, Vuolteenaho K, Nieminen R, Moilanen T, Moilanen E (2011) Leptin enhances MMP-1, MMP-3 and MMP-13 production in human osteoarthritic cartilage and correlates with MMP-1 and MMP-3 in synovial fluid from OA patients. Clin Exp Rheumatol 29(1): 57-64.

17. Lajeunesse D, Pelletier JP, Martel-Pelletier J (2005) Osteoarthritis: a metabolic disease induced by local abnormal leptin activity? Curr Rheumatol Rep 7(2): 79-81.

18. Miller GD, Nicklas BJ, Davis CC, Ambrosius WT, Loeser RF, et al. (2004) Is serum leptin related to physical function and is it modifiable through weight loss and exercise in older adults with knee osteoarthritis? Int J Obes Relat Metab Disord 28(11): 1383-1390.

19. Mutabaruka MS, Aoulad Aissa M, Delalandre A, Lavigne M, Lajeunesse D (2010) Local leptin production in osteoarthritis subchondral osteoblasts may be responsible for their abnormal phenotypic expression. Arthritis Res Ther 12(1): R20.

(5) Not relevant to leptin: 22

1. Beekhuizen M, Gierman LM, van Spil WE, Van Osch GJ, Huizinga TW, et al. (2013) An explorative study comparing levels of soluble mediators in control and osteoarthritic synovial fluid. Osteoarthritis Cartilage 21(7): 918-922.

2. de Boer TN, van Spil WE, Huisman AM, Polak AA, Bijlsma JW, et al. (2012) Serum adipokines in osteoarthritis; comparison with controls and relationship with local parameters of synovial inflammation and cartilage damage. Osteoarthritis Cartilage 20(8): 846-853.

3. Gegout PP, Francin PJ, Mainard D, Presle N (2008) Adipokines in osteoarthritis: friends or foes of cartilage homeostasis? Joint Bone Spine 75(6): 669-671.

4. Karvonen-Gutierrez CA, Sowers MR, Heeringa SG (2012) Sex dimorphism in the association of cardiometabolic characteristics and osteophytes-defined radiographic knee osteoarthritis among obese and non-obese adults: NHANES III. Osteoarthritis Cartilage 20(7): 614-621.

5. Perruccio AV, Mahomed NN, Chandran V, Gandhi R (2014) Plasma adipokine levels and their association with overall burden of painful joints among individuals with hip and knee osteoarthritis. J Rheumatol 41(2): 334-337.

6. Staikos C, Ververidis A, Drosos G, Manolopoulos VG, Verettas DA, et al. (2013) The association of adipokine levels in plasma and synovial fluid with the severity of knee osteoarthritis. Rheumatology (Oxford) 52(6): 1077-1083.

7. ai MF, Sandell LJ (2011) Inflammatory mediators: tracing links between obesity and osteoarthritis. Crit Rev Eukaryot Gene Expr 21(2): 131-142.

8. Anandacoomarasamy A, Giuffre BM, Leibman S, Caterson ID, Smith GS, et al. (2009) Delayed gadolinium-enhanced magnetic resonance imaging of cartilage: clinical associations in obese adults. J Rheumatol 36(5): 1056-1062.

9. Berry PA, Jones SW, Cicuttini FM, Wluka AE, Maciewicz RA (2011) Temporal relationship between serum adipokines, biomarkers of bone and cartilage turnover, and cartilage volume loss in a population with clinical knee osteoarthritis. Arthritis Rheum 63(3): 700-707.

10. Conde J, Scotece M, Lopez V, Gomez R, Lago F, et al. (2013) Adipokines: novel players in rheumatic diseases. Discov Med 15(81): 73-83.

11. Ding C, Jones G, Wluka AE, Cicuttini F (2010) What can we learn about osteoarthritis by studying a healthy person against a person with early onset of disease? Curr Opin Rheumatol 22(5): 520-527.

12. Distel E, Cadoudal T, Durant S, Poignard A, Chevalier X, et al. (2009) The infrapatellar fat pad in knee osteoarthritis: an important source of interleukin-6 and its soluble receptor. Arthritis Rheum 60(11): 3374-3377.

13. Gabay O, Hall DJ, Berenbaum F, Henrotin Y, Sanchez C (2008) Osteoarthritis and obesity: experimental models. Joint Bone Spine 75(6): 675-679.

14. Gandhi R, Santone D, Takahashi M, Dessouki O, Mahomed NN (2013) Inflammatory predictors of ongoing pain 2 years following knee replacement surgery. Knee 20(5): 316-318.

15. Gandhi R, Takahashi M, Rizek R, Dessouki O, Mahomed NN (2012) Obesity-related adipokines and shoulder osteoarthritis. J Rheumatol 39(10): 2046-2048.

16. Gandhi R, Takahashi M, Virtanen C, Syed K, Davey JR, et al. (2011) Microarray analysis of the infrapatellar fat pad in knee osteoarthritis: relationship with joint inflammation. J Rheumatol 38(9): 1966-1972.

17. Gegout PP, Francin PJ, Mainard D, Presle N (2008) Adipokines in osteoarthritis: friends or foes of cartilage homeostasis? Joint Bone Spine 75(6): 669-671.

18. Heidari B (2011) Knee osteoarthritis prevalence, risk factors, pathogenesis and features: Part I. Caspian J Intern Med 2(2): 205-212.

19. Ibrahim SM, Hamdy MS, Amer N (2008) Plasma and synovial fluid adipocytokines in patients with rheumatoid arthritis and osteoarthritis. Egypt J Immunol 15(1): 159-170.

20. Iliopoulos D, Malizos KN, Tsezou A (2007) Epigenetic regulation of leptin affects MMP-13 expression in osteoarthritic chondrocytes: possible molecular target for osteoarthritis therapeutic intervention. Ann Rheum Dis 66(12): 1616-1621.

21. Issa RI, Griffin TM (2012) Pathobiology of obesity and osteoarthritis: integrating biomechanics and inflammation. Pathobiol Aging Age Relat Dis 2(2012).

22. Jiang LS, Zhang ZM, Jiang SD, Chen WH, Dai LY (2008) Differential bone metabolism between postmenopausal women with osteoarthritis and osteoporosis. J Bone Miner Res 23(4): 475-483.

(6) Not relevant to osteoarthritis: 25

1. Pupek-Musialik D, Musialik K, Hen K (2010) Obesity--a challenge for modern ortopedy. Chir Narzadow Ruchu Ortop Pol 75(4): 236-241.

2. Wang ZH, Zhang P, Jiao WX, Ren ZQ, Wang TJ (2007) The significance of the detection of serum leptin in rheumatoid arthritis. Med Information 20(7): 1222-1223.

3. Wislowska M, Rok M, Jaszczyk B, Stepien K, Cicha M (2007) Serum leptin in rheumatoid arthritis. Rheumatol Int 27(10): 947-954.

4. Wang ZH, Zhang P, Jiao WX, Ren ZQ, Wang TJ (2007) The significance of the detection of serum leptin in rheumatoid arthritis. Med Information 20(7): 1222-1223.

5. Clockaerts S, Bastiaansen-Jenniskens YM, Feijt C, De Clerck L, Verhaar JA, et al. (2012) Cytokine production by infrapatellar fat pad can be stimulated by interleukin 1beta and inhibited by peroxisome proliferator activated receptor alpha agonist. Ann Rheum Dis 71(6): 1012-1018.

6. Karvonen-Gutierrez CA, Harlow SD, Jacobson J, Mancuso P, Jiang Y (2014) The relationship between longitudinal serum leptin measures and measures of magnetic resonance imaging-assessed knee joint damage in a population of mid-life women. Ann Rheum Dis 73(5): 883-889.

7. Liang J, Feng J, Wu WK, Xiao J, Wu Z, et al. (2011) Leptin-mediated cytoskeletal remodeling in chondrocytes occurs via the RhoA/ROCK pathway. J Orthop Res 29(3): 369-374.

8. Loeser RF (2003) Systemic and local regulation of articular cartilage metabolism: where does leptin fit in the puzzle? Arthritis Rheum 48(11): 3009-3012.

9. Magrans-Courtney T, Wilborn C, Rasmussen C, Ferreira M, Greenwood L, et al. (2011) Effects of diet type and supplementation of glucosamine, chondroitin, and MSM on body composition, functional status, and markers of health in women with knee osteoarthritis initiating a resistance-based exercise and weight loss program. J Int Soc Sports Nutr 8(1): 8.

10. Scotece M, Conde J, Gomez R, Lopez V, Lago F, et al. (2011) Beyond fat mass: exploring the role of adipokines in rheumatic diseases. ScientificWorld Journal 11(1932-1947.

11. Senolt L, Housa D, Vernerova Z, Jirasek T, Svobodova R, et al. (2007) Resistin in rheumatoid arthritis synovial tissue, synovial fluid and serum. Ann Rheum Dis 66(4): 458-463.

12. Stannus OP, Cao Y, Antony B, Blizzard L, Cicuttini F, et al. (2013) Cross-sectional and longitudinal associations between circulating leptin and knee cartilage thickness in older adults. Ann Rheum Dis.

13. Tong KM, Shieh DC, Chen CP, Tzeng CY, Wang SP, et al. (2008) Leptin induces IL-8 expression via leptin receptor, IRS-1, PI3K, Akt cascade and promotion of NF-kappaB/p300 binding in human synovial fibroblasts. Cell Signal 20(8): 1478-1488.

14. Toussirot E, Streit G, Wendling D (2007) The contribution of adipose tissue and adipokines to inflammation in joint diseases. Curr Med Chem 14(10): 1095-1100.

15. Schaffler A, Ehling A, Neumann E, Herfarth H, Tarner I, et al. (2003) Adipocytokines in synovial fluid. JAMA 290(13): 1709-1710.

16. Wang ZH, Zhang P, Jiao WX, Ren ZQ, Wang TJ (2007) The significance of the detection of serum leptin in rheumatoid arthritis. Med Information 20(7): 1222-1223.

17. Stannus OP, Cao Y, Antony B, Blizzard L, Cicuttini F, et al. (2013) Cross-sectional and longitudinal associations between circulating leptin and knee cartilage thickness in older adults. Ann Rheum Dis.

18. Tong KM, Shieh DC, Chen CP, Tzeng CY, Wang SP, et al. (2008) Leptin induces IL-8 expression via leptin receptor, IRS-1, PI3K, Akt cascade and promotion of NF-kappaB/p300 binding in human synovial fibroblasts. Cell Signal 20(8): 1478-1488.

19. Toussirot E, Streit G, Wendling D (2007) The contribution of adipose tissue and adipokines to inflammation in joint diseases. Curr Med Chem 14(10): 1095-1100.

20. Schaffler A, Ehling A, Neumann E, Herfarth H, Tarner I, et al. (2003) Adipocytokines in synovial fluid. JAMA 290(13): 1709-1710.

21. Pupek-Musialik D, Musialik K, Hen K (2010) Obesity--a challenge for modern ortopedy. Chir Narzadow Ruchu Ortop Pol 75(4): 236-241

22. Wang ZH, Zhang P, Jiao WX, Ren ZQ, Wang TJ (2007) The significance of the detection of serum leptin in rheumatoid arthritis. Med Information 20(7): 1222-1223.

23. Wislowska M, Rok M, Jaszczyk B, Stepien K, Cicha M (2007) Serum leptin in rheumatoid arthritis. Rheumatol Int 27(10): 947-954.

24. Clockaerts S, Bastiaansen-Jenniskens YM, Feijt C, De Clerck L, Verhaar JA, et al. (2012) Cytokine production by infrapatellar fat pad can be stimulated by interleukin 1beta and inhibited by peroxisome proliferator activated receptor alpha agonist. Ann Rheum Dis 71(6): 1012-1018.

25. Conde J, Scotece M, Lopez V, Gomez R, Lago F, et al. (2013) Adipokines: novel players in rheumatic diseases. Discov Med 15(81): 73-83.
